# The Ethics of Human–Animal Relationships and Public Discourse: A Case Study of Lions Bred for Their Bones

**DOI:** 10.3390/ani9020052

**Published:** 2019-02-08

**Authors:** Peter Coals, Dawn Burnham, Andrew Loveridge, David W. Macdonald, Michael ’t Sas-Rolfes, Vivienne L. Williams, John A. Vucetich

**Affiliations:** 1 Wildlife Conservation Research Unit, Recanati-Kaplan Centre, Department of Zoology, University of Oxford, Tubney OX13 5QL, UK; dawn.burnham@zoo.ox.ac.uk (D.B.); andrew.loveridge@zoo.ox.ac.uk (A.L.); david.macdonald@zoo.ox.ac.uk (D.W.M.); vivienne@wildscience.co.za (V.L.W.); 2 School of Animal, Plant & Environmental Science, University of the Witwatersrand, Johannesburg 2000, South Africa; tsas.rolfes@gmail.com; 3 School of Geography and the Environment, University of Oxford, Oxford OX1 3QY, UK; 4 School of Forest Resources and Environmental Science, Forestry and Wood Products Building, 1400 Townsend Drive, Houghton, MI 49931-1295, USA

**Keywords:** *Panthera leo*, traditional medicine, cultural value, intrinsic value, conservation ethics, wildlife trade, captive lion hunting, captive lion breeding

## Abstract

**Simple Summary:**

In South Africa, lions are farmed, and a product of that farming is lion skeletons that form part of an international trade to supply traditional medicine markets in Southeast Asia with felid bones. As a matter of public policy, the practice is a complicated nexus of concerns for entrepreneurial freedom, wildlife conservation, and the fair treatment of animals. We used this case to demonstrate how public discourse about ethically-charged policies can be aided by a technique from the academic field of applied ethics, i.e., formal argument analysis. We showed how the technique can be integrated into existing frameworks for public decision-making. To further facilitate the application of this technique to other cases, we also offered ten general lessons for formal analysis of ethical arguments.

**Abstract:**

Conservation and natural resource management are increasingly attending the ethical elements of public decisions. Ethical considerations are challenging, in part, because they typically require accounting for the moral consideration of various human and nonhuman forms of life, whose interests sometimes conflict (or seem to conflict). A valuable tool for such evaluations is the formal analysis of ethical arguments. An ethical argument is a collection of premises, logically interrelated, to yield a conclusion that can be expressed in the form, “*We ought to…*” According to the rules of logic, a conclusion is supported by an argument if all its premises are true or appropriate and when it contains no mistaken inferences. We showed how the formal analysis of ethical arguments can be used to engage stakeholders and decision-makers in decision-making processes. We summarised the method with ten specific guidelines that would be applicable to any case. We illustrated the technique using a case study focused on captive-bred lions, the skeletons of which form part of an international trade to supply traditional medicine markets in Southeast Asia with felid bones. As a matter of public policy, the practice is a complicated nexus of concerns for entrepreneurial freedom, wildlife conservation, and the fair treatment of animals.

## 1. Introduction

Public decisions pertaining to relationships between humans and other animals routinely involve transdisciplinary synthesis of ideas that spans a breath-taking range of knowledge—ecology, organismal sciences, sciences pertaining to human behavior, politics, economics, ethics, and more [1,2,3].

Heaped upon that challenge are different parties engaged in the decision-making process who routinely have different understandings of the various empirical and normative claims that pertain to a decision, and sometimes even a different understanding of the relevance of various claims to the decision. More frustrating, the parties often inadequately understand the limits of their agreements and disagreements with other engaged parties. That inadequacy is a dreadful hindrance to discovery of common ground that can lead to a wise decision—whether that common ground be made via discovery of perspectives that transcend differences or through one party persuading a decision-maker of the limits of a perspective held by another party.

Scientific uncertainty is often raised as another complication. And it is. Yet often the more pernicious complications are differences in the ethical values among parties to a decision-making process. The challenge here may not be so much whether an engaged party acknowledges the moral value of nonhuman organisms or the collectives they form—populations, species, and ecosystems. The greater challenge rises from differences in judgment about how to adjudicate conflicts between different subjects of moral concern: Under what circumstances, if any, do the interests of some humans outweigh the interests of some nonhumans? Under what circumstances, if any, do the interests of individual animals outweigh the interests of entire populations and species?

On those questions, ethicists have offered bookshelves’ worth of analysis. The varied positions taken by the most thoughtful and robust analysis—representing, for example, animal ethics [4] and conservation ethics [5]—suggest that critical issues remain unresolved by ethicists. Moreover, the engaged parties often hold strong intuitions on these matters, and many are not well equipped to evaluate those intuitions in an environment replete with countervailing intuitions.

There is a need for a mode of discourse suited to these challenges—especially the challenge of conducting ethical discourse with non-ethicists. To that end, we consider a basic tool of applied ethics, i.e., argument analysis. We make no pretense that this tool is sufficient for handling the abovementioned challenges, but it may well be necessary. This tool is, for example, especially well-suited to synthesising disparate forms of knowledge, exposing important but subtle points of agreement and disagreement, and exposing limitations that may lurk beneath any position.

We demonstrate this mode of discourse in the context of a case study concerning lions. We describe the case study (Section 2), describe the basic outlines of argument analysis (Section 3), and then demonstrate its application to the case (Section 4, Section 5, Section 6 and Section 7). In doing so, we identified several general lessons that are applicable far beyond the case examined here.

## 2. The Case Example

Humans’ relationship with lions is a prime example of raising concerns about both conservation and animal welfare. Lions are raised and bred in commercial farming facilities around the world for a variety of uses, including trophy hunting, cub petting, wildlife viewing, and other forms of entertainment and education. In South Africa, there are more than 400 registered facilities for breeding and rearing at least 7800 lions [6,7]. Since 2008, farmed lions from South Africa have also been used for an additional purpose. In particular, after the lions are killed, their skeletons and other body parts (mainly fangs and claws) are harvested and legally traded to Southeast Asia, where these skeletal materials are used as a substitute for, or in addition to, tiger (*Panthera tigris*) bones in traditional Asian medicine, health tonics, and ornaments [6]. Alongside the Asian demand, there is also a demand within Africa for lion parts for use in zootherapeutic (animal-based) traditional medicines and other cultural practices [8]. These emerging uses of lion bones are central to the case example.

These emerging uses are also directly relevant to an international treaty focused on the conservation of endangered species, i.e., the Convention on International Trade in Endangered Species of Wild Fauna and Flora (CITES) [9,10]. CITES and its 183 signatory nations (Parties) presuppose that wildlife trade is appropriate, in principle [11]. CITES also acknowledges that international trade in wildlife can endanger or exacerbate the endangerment of certain species, unless that trade is strictly regulated. South Africa is a party to CITES and African lions are (one of about 21,000 species) listed in Appendix II of CITES. Those circumstances mean that South Africa is obligated to set an annual quota for the number of lion skeletons permitted to leave their country.

CITES requires the quota must be set below a level that would otherwise further endanger the persistence of *Panthera leo*, the species [11]. For context, lions have disappeared from 92% of their historic range and have declined by 43% during the two decades between 1993 and 2014, leaving an estimated 23,000–39,000 individuals living in the wild [12]. Declines are expected to continue and are in large part due to prey depletion, livestock encroachment, illegal killing due to conflict over livestock, and habitat conversion.

In total, over 6000 skeletons have been permitted under CITES for export from South Africa to Southeast Asia since 2008, peaking at more than 1700 skeletons in 2016 [13]. In 2017, a quota of 800 skeletons was introduced. In July 2018, the quota amount was raised to 1500; however, in December 2018, that figure was revised to 800. Aside from volatility in the quota, concern about the quota will almost certainly persist. Recent and upcoming negotiations surrounding these quotas have sparked a new dimension to discourse about the use of captive lions. The maximum allowable quota, under CITES, is supposed to be determined by conservation science. The science is often incomplete, politicised (by those favouring higher and lower quotas), and subsequently negotiated [11]. As a sovereign nation, South Africa is also allowed to set a quota lower than the maximum allowable for whatever reason it may so decide.

The subject of this analysis focuses on an activity for which there is no neutral vocabulary. That is, opponents refer to “lion farming” and supporters are more likely to refer to “captive-bred lions”. Acknowledging that there is no neutral expression for these ideas, we refer hereafter to this activity as lion farming.

We contribute insight to this discourse using argument analysis, a basic method of applied ethics which aims to better understand reasons for how we ought to behave. Argument analysis has been used to contribute insight to other conservation-related issues [14]. Our intention is not to completely resolve issues pertaining to the use of farmed lions for their bones. The issue is too new and unexplored to permit a robust treatment in a single effort. Rather, our intent is to find the approximate outlines for the landscape of considerations required for robust treatment of the issue.

## 3. Argument Analysis

An argument is a collection of premises intended to support some conclusion [15]. An ethical argument is one whose conclusion can be expressed in the forms “*We should…*” or “*We should not…*”. An ethical argument, like any kind of argument, supports its conclusions when it is sound and valid; that is, when all its premises are true or appropriate and when it contains no mistaken inferences [16,17].

A first step in argument analysis is to express a reason—i.e., an informal and typically incompletely expressed justificatory reason—for behaving in a certain way; the second step is to express that reason as a formal argument. A third step is to evaluate the argument. This evaluation includes making judgements about the appropriateness of stated premises.

An argument fails to support its conclusion if any of the following conditions do not hold: (1) Any stated premise is inappropriate, (2) the conclusion depends on an unstated premise that is inappropriate, or (3) the argument is invalid—meaning the premises may be true, but they do not lead to the conclusion [15]. That an argument is unsound (or invalid) is not definitive proof that the conclusion of that argument is wrong, but it does mean that the conclusion is not supported by that given argument.

Ethical arguments are like ecological models in the sense that their value lies not in being perfect, complete representations of the world [18]. Rather, their value lies in the potential for revealing insights that might otherwise be overlooked. A value of argument analysis for public discourse is its clarity, precision, and transparency. For examples of argument analysis in the conservation literature see References [14] and [19].

In Section 4, Section 5, Section 6 and Section 7, we present four sets of arguments that represent the dominant topics of discourse on South Africa’s quotas for exporting lion skeletons. Given that the issue is driven by CITES, two of the four arguments pertain to conservation. One of the arguments is focused on economics and another on the fair treatment of individual animals. Even though the central concerns of the last two arguments are beyond the interest of CITES, those concerns have varying degrees of influence in the presence of uncertain or politicised conservation science [11].

We assessed these arguments individually, because many of the engaged stakeholders have one primary concern. As such, it is useful to develop individual arguments motivated by each of those concerns, so stakeholders see clearly how each concern is evaluated. Because the concerns are interrelated, the arguments are distinct but not wholly independent. Ultimately, there is a need to develop synthetic insight from the arguments considered as a set. That is best left as a subsequent step of analysis because the simpler, individual arguments often strain the ability of many engaged parties, given their limited experience with argument analysis. These considerations are congruent with thinking of ethical arguments as heuristic models of considerable value, rather than perfectly complete representations. In Section 8, we offer thoughts about how to implement this mode of communication in public discourse.

Finally, we use “lion farming”, hereafter, as shorthand for “farming lions with use of their bones”. We focus on lion bones, as opposed to other uses of farmed lions, for reasons outlined in Section 2. While many of the insights that follow are likely relevant to farming lions for other purposes, that determination is beyond the scope of this paper.

## 4. Conservation-Focused Arguments and Empirical Uncertainty

Here, we present a set of arguments in support of lion farming, followed by a set that oppose lion farming. These arguments also illustrate how uncertainty about empirical claims (premises) affects the analysis of ethical arguments.

### 4.1. Arguments in Support of Lion Farming

Several reasons offered in support of lion farming focus on purported benefits to various facets of conservation. Each of these reasons can be expressed as an argument whose generalised structure is:

- Premise 1 (P1). Lion farming promotes some aspect of conservation.
- Premise 2 (P2). We ought to promote conservation.
- Conclusion 1 (C1). Lion farming is appropriate.

Think of P1 as a placeholder for more specific expressions of P1. These more specific expressions of P1, all beginning “Lion farming…”, include:

- P1(a). …prevents conversion of land to uses less valuable for biodiversity.
- P1(b). …lessens the risk of overexploiting wild populations of lions.
- P1(c). …lessens the risk of overexploiting wild felids, especially tigers.
- P1(d). …could contribute to conserving the genetic diversity of lions.

An important element of evaluating these arguments would be empirical evaluation of the claims represented by P1(a–d). To that end, we present some comments on each premise in the paragraphs that follow. These comments are not intended to be definitive, only indicative of the issues that would arise if one were to provide an adequate evaluation.

#### 4.1.1. Premise P1(a)

It is possible that lion farming contributes substantively to the maintenance of biodiversity by supplying lions for captive-bred hunts that take place on lands also inhabited by many forms of biodiversity. One might argue that those lands would be converted to uses less suitable for maintaining biodiversity if the supply of farmed lions was diminished. One of several considerations for evaluating this line of thought is: While private wildlife hunting and ranching lands in South Africa cover over 16% of total land area [20], not all of that 16% is used for captive-bred lion hunts, and most of these hunting areas are fenced and small, averaging approximately 3000 ha [7]. Ultimately, the truth-value of premise P1(a) is not known and might be fairly characterised as far from certain. A second consideration for evaluating this argument is whether the emerging use of lions for their bones would affect the motivation to protect habitat on lands where farmed lions are trophy-hunted.

#### 4.1.2. Premise P1(b)

Overexploitation (legal and illegal killing) is a significant threat to a number of (though not all) wild populations of lions [21]. Demonstrating the truth of P1(b) would likely require showing that lion farming (in conjunction with captive-bred lion hunts) prevents substantive increases in human-caused mortality in those wild populations of lions where overexploitation is a threat. To offer a reasonably robust demonstration on this matter would be no small feat. Ultimately, the truth-value of P1(b) is uncertain and is likely to remain so for the foreseeable future.

#### 4.1.3. Premise P1(c)

Wild tiger conservation is threatened by overexploitation, motivated in part by a consumer demand for tiger bones, especially in Southeast Asia [13]. It is possible that the provision of a legal supply of lion bones might act as a partial substitute to satisfy tiger bone demand that might otherwise be met from illegal sources of tiger products. The correct technical argument would be that the legal supply of lion bones may act to suppress prices for illegally provided wild tiger bones, thereby reducing incentives for wild tiger poaching. However, it is also plausible that the legal supply of lion bones would fuel demand for illegally obtained bones of wild felids [8,22]. If so, it would not be the first time that market pressures expected to favour conservation led to unintended detriments to conservation [23]. In any case, we explore this countervailing idea below (i.e., argument P3, P4, C2). Ultimately, the truth-value of P1(c) is uncertain and is likely to remain so for the foreseeable future.

#### 4.1.4. Premise P1(d)

There are instances where captive and privately-owned wildlife have contributed substantively to the demographic and genetic restoration of wild populations [24], such as with the Arabian oryx [25]. A prerequisite for using farmed lions to maintain the genetic health of wild lions would be an adequate understanding of the genetics of farmed lions (e.g., a reliably maintained studbook). Moreover, the number of wild lions and wild lion populations across Africa is sufficiently large (ca. 23,000–39,000 lions in approximately 65 populations), such that the genetic health of wild lions is likely best maintained by connectivity (human-assisted or otherwise) among existing populations of wild lions [26,27]. Premise P1(d) is unlikely to be true for the foreseeable future.

### 4.2. Reminder

A complete evaluation of the aforementioned premises (and the premises to follow) is beyond the scope of this piece. Here, our aim is to outline some of the key issues that require evaluation. A more complete assessment of the limits of empirical knowledge as it pertains to farming lions with use of their bones is the subject of a forthcoming analysis [28].

### 4.3. Arguments that Oppose Lion Farming

Another conservation-focused argument indicates that lion farming should be opposed. This argument represents, in a sense, the antithesis of premises P1(b) and P1(c) from the previous section. The argument is:

- Premise 3 (P3). Lion farming stimulates a demand for bones from wild sources of endangered felids—especially lions, tigers, leopards, and jaguars—that threatens the conservation of such felids.
- Premise 4 (P4). We ought not threaten the conservation of endangered felids.
- Conclusion 2 (C2). Lion farming is inappropriate.

(We intend the argument represented by P1, P2, and C1 to be separate from this argument, represented by P3, P4, and C2. That is, this second argument is not a continuation of the first. We assign each premise in this paper with its own number to prevent confusion when we refer to various premises in the main text.)

A general concern among conservationists is that legal trade involving an endangered species may facilitate or even exacerbate illegal trade that imperils the endangered species [29]. This concern is grounded in economic theory, which outlines various conditions under which wildlife farming and associated legal trade may either support or undermine conservation goals [30,31]. However, there is relatively little decisive empirical evidence on the subject. Important examples include orchids, elephant tusks, and tiger bones [32,33,34]. With tiger bones, the concern has been that legal trade of bones derived from captive-bred tigers in China might promote an illegal trade in bones derived from tigers poached in the wild [34]. With respect to lion bones, the concern is twofold. First, legally traded lion bones might promote illegal trade in bones from lions poached in the wild. Second, legally traded lion bones might promote illegal trade in the bones of other endangered felids [35]. The second concern rises from observing that lion bones and those of other felid species may act as substitutes for one another in trade, complicating enforcement efforts and possibly enabling trade in those other species. An added concern is that illegal trade in endangered species supports transnational organised crime that also traffics humans, guns, and drugs [36,37]. While these concerns merit attention, little substantiated evidence exists to support or refute them. Nevertheless, any additional strain may worsen the already perilous conservation status of such felids [38]. Ultimately, the truth-value of P1(c) is uncertain and is likely to remain so for the foreseeable future.

### 4.4. General Lessons

The analysis thus far represents a common feature of analysing ethical arguments. Ethical arguments typically comprise both ethical premises (sometimes called normative premises) and empirical premises (sometimes called descriptive premises). In the preceding arguments, the normative premises are P2 and P4; the empirical premises are P1 and P3. Ethical premises are typically evaluated with ethical reasoning and empirical premises are evaluated with scientific evidence. Also recall that an argument fails to support its conclusion if even one premise—including an empirical premise—is inappropriate.

The first general lesson (Table 1) from this analysis is that many controversies in conservation ethics are presumed to depend on underlying, intractable ethical premises [39,40,41,42]. Our analysis illustrates an important, and likely common, circumstance: Ethical conclusions can fail to be supported because empirical premises do not hold. This circumstance is an antidote to a common misconception about ethics, the misconception being: Evaluating ethical arguments necessarily requires evaluating underlying, thorny, and seemingly intractable ethical premises. Often enough, ethical analysis may be resolved by evaluating empirical claims.

A second general lesson pertains to the evaluation of an ethical argument comprising empirical premises whose truth-values are unknown. One way to account for uncertainty in a deductive argument is to adjust key wording in the argument. For example, reconsider the argument:

- P1. Lion farming promotes some aspect of conservation.
- P2. Conservation ought to be promoted.
- C1. Lion farming ought to be promoted.

Suppose available scientific evidence indicates that P1 is “about as likely as not” to be true. This uncertainty can be incorporated into the premise, resulting in a premise whose truth-value is simply true:

- P1′. It is about as likely as not that lion farming promotes some aspect of conservation

This change to P1 necessitates a change to the conclusion:

- C1′. It is about as likely as not that lion farming is appropriate.

In other words, the uncertainty of a premise is inherited by any subsequent conclusion.

Scientific evaluations are often precisely and quantitatively probabilistic. By contrast, deductive reasoning is an essentially qualitative endeavour. Useful guidance for bridging that gap is represented by language adopted by the Intergovernmental Panel on Climate Change (IPCC), which associates levels of certainty with English phrases that can readily be incorporated into the premises and conclusions of a deductive argument (Table 2).

When an argument offers inconclusive support for an ethical claim, especially due to uncertainty in an empirical premise, one might consider invoking the precautionary principle to solve what otherwise would seem an impasse. The precautionary principle refers roughly to the notion of refraining from actions that carry a risk of irreparable harm. Asserting the precautionary principle onto an inconclusive argument would lead to either accepting various premises (such as P1 or P3) as true until demonstrated otherwise, or vice versa. However, knowing where to set that burden of proof would depend just about entirely on one’s perception about what counts as an irreparable harm. In our case, one could primarily see irreparable harm to farmed lions, or to the conservation of wild populations of lions, or to lion farmers (economic harm, see below). Proper application of the precautionary principle to argument analysis (when used to gain insight about resolving conflicts among parties who see an issue differently) is to build and evaluate a separate argument that can offer insight about which irreparable harm deserves the most precaution. Other challenges associated with invoking the precautionary principle are highlighted in References [44] and [45]. In summary, a third general lesson to draw from our analysis is that invoking the precautionary principle will not typically offer a quick solution to an impasse rising from premises with uncertain truth-value; however, an impasse may be dissolved with careful evaluation of which irreparable harm deserves the most precaution (Table 1).

## 5. Economic-Focused Arguments, Ends, and Means

Here, we consider arguments focusing on economic facets of lion farming. Doing so continues to illustrate the breadth of issues associated with lion farms, as well as illustrating general lessons about the analysis of ethical arguments—i.e., lessons about handling conflicts between purportedly justifiable ends and purportedly objectionable means for realising those ends. Consider this argument focused on economics:

- Premise 5 (P5). Lion farming generates income and maintains jobs and other benefits to the economy.
- Premise 6 (P6). We should promote benefits to the economy.
- Conclusion 3 (C3). Therefore, we ought to promote lion farming.

Premise P5 is like P1 in being an empirical premise, and the truth-value of P5 would be evaluated by formal economic analysis—akin to the kind of analysis provided for fox hunting in the UK [46,47]. P5 is also like P1 in that its evaluation is greatly aided by expressing the relevant facets of the economy in sufficiently precise terms. In this vein, the phrase “other benefits to the economy” in P5 is inappropriately imprecise. That phrase should be replaced with appropriately specific elements of the economy or omitted from P5.

P6, a normative premise, represents a goal or desired end, i.e., an economy that adequately serves its people. The implication of P6 (when joined to the conclusion, C3) is developing or maintaining such an economy requires the income and jobs associated with lion farming. An argument focused on economics—if it is to be a sound and valid argument—needs premises with an adequately precise account of the end-goals of a healthy economy (e.g., How many jobs should exist in a given population? How much income should be generated in an economy, and how ought that income be distributed?). For more on the broader importance of properly specifying the end-goals associated with economics, see Raworth (2017) [48].

Furthermore, arguments focused on ends may also need to attend to the means used to achieve the proffered end-goal: Is lion farming an acceptable means for realising the expressed economic goals, vaguely expressed as they are in P5? Questions about whether ends justify means are among the thorniest questions in ethics, beseeching a variety of sophisticated considerations. Here, we point to some of the most important and then refer readers to additional sources.

First, the issue is not resolved by merely asserting principles of utilitarianism—a framework in academic ethics that might seem to assert (in a case like this) that the means are justified. The assertion would be unsatisfying for being an assertion rather than a demonstration of why those principles are apt to this situation. It would be similarly unsatisfying to merely assert principles of deontology, a contrasting framework that might seem to assert (in a case like this) that the means are not justified.

A robust treatment of this issue would demonstrate (e.g., via argument analysis), as opposed to merely asserting, how principles of utilitarianism or deontology ought (or ought not) to be applied. For example, utilitarianism is a kind of consequentialism; as such, its use is limited to cases where one has an adequate capacity to predict the consequences of the action(s) in question. Limited capacity for prediction is a hallmark of developing policy associated with economics and human–nature relationships. Any claim to employ the principles of utilitarianisms should meet that challenge.

Deontology has its own challenges to meet. For example, deontology supports actions that fulfil one’s duties to another and rejects actions that conflict with those duties—even if the duty-conflicting actions seem to lead to a benevolent end-goal. A challenge for deontology is to identify all the relevant duties and adjudicate conflicts among them. To illustrate the challenge, one’s duty to be truthful and kind are often both relevant to a particular situation and they sometimes conflict, as when being kind seems to call for being untruthful (such as white lies). A similar circumstance swirls round one’s duty to honour another’s freedom and our duty to act in the interest of safety. Archetypal examples include restricting the freedom of a mentally-impaired person who is a threat to safety and protecting national security by restricting citizens’ freedoms. Each case is always adjudicated, and the adjudication is reflected by the action taken. The unrelenting question is, what makes for a fair adjudication. For one perspective on fair adjudication and an introduction to other sources on the subject, see Reference [49].

Finally, to limit one’s self to either the principles of utilitarianism or deontology often lends less resolution than is sometimes supposed [50,51]. For example, many end-goals can be re-expressed as duties and vice versa. More precisely, a deontologist may oppose lion farming because it violates a duty to treat lions in a certain manner; but another deontologist might support lion farming because it fulfils a duty to allow lion farmers the freedom to make a livelihood of their choosing. Conversely, a consequentialist could support lion farming in the belief that it will result in conservation benefits; but another consequentialist might oppose lion farming on grounds that we ought to maximise the beneficial outcomes, not so much to populations or species, but first and foremost to individual animals (humans and nonhumans).

These considerations are not conjured ex nihilo. Following the lead of Singer (1975) [52], for example, one would oppose lion farming on utilitarian grounds—even though a supporter of lion farming might also use utilitarian principles to explain their perspective. The ideas of Nussbaum (2006) [53] would seem to lead to opposition of lion farming—not so much by the general principles of deontology, but rather from taking for granted that duties to individual organisms outweigh duties to ecological collectives. By contrast, the postulates of conservation in Soulé (1985) [54]—which include that biotic diversity is good and extinction should be avoided—could (we suppose) be used in a deontological argument in support of lion farming—so long as it supported the goals of conservation.

Again, resolution is not determined by siding with deontology or utilitarianism. No less important for resolving the issue is to understand who deserves precisely what moral consideration and how those considerations ought to be reconciled when they conflict.

A fourth and a fifth general lesson emerges from the analysis of this economic-focused argument. The fourth lesson is a need to attend not only to the consequences of an action, but also to concerns about whether the consequences can be justified given the proffered means. Proper tending of this issue is essential and difficult, though workable.

The fifth lesson is that evaluating claims about ends and means is greatly aided by expressing the end-goal with sufficient precision (e.g., a vague end-goal of “create new jobs” might be expressed more precisely as “decrease unemployment rate in [*insert name of some geographic region or group of people*] to less than 5%”). Specificity is an aid in evaluating (1) the merit of the end-goal, (2) alternative means of realising the end-goal, and (3) whether the proffered means would lead substantively (or only trivially) to the end-goal.

Application of those lessons to the case of lion farming leads to: If a proponent for farming is honestly and primarily motivated by economic concern (as is implied by the arguments P5, P6, and C3), then it is appropriate to ask, of the various means by which an appropriately specific end-goal (perhaps, gainful employment) might be realised, which competes the least with other genuine interests? The answer is likely to offer significant insight about the appropriateness of lion farming.

While these general lessons are illustrated by the economics-focused argument, they are equally applicable to, for example, conservation-focused arguments. It is appropriate to ask, more precisely, what the goals of lion conservation are and if a prohibition (or allowance of) lion farming is an appropriate means of realising those goals [18].

## 6. Intrinsic Value

Some may intuit that lion farming is wrong because lions possess intrinsic value and for that reason should not be farmed. The formal argument associated with that reasoning might be expressed as:

- Premise 7 (P7). Individual lions possess intrinsic value.
- Premise 8 (P8). Possessors of intrinsic value should be treated fairly and with concern for their interests.
- Premise 9 (P9). Farming is not a fair way to treat lions.
- Conclusion 4 (C4). Lions should not be subjected to farming.

The argument is readily and usefully generalised:

- P7′. X possesses intrinsic value.
- P8. Possessors of intrinsic value should be treated fairly and with concern for their interests.
- P9′. Y is not a fair way to treat X.
- C4′. X should not be subjected to Y.

The generalised form of the argument quickly leads to realisation that X could stand for lions, lion farmers or citizens who oppose lion farming because they prefer to live in a society without lion farming. And Y could be replaced with any unfair treatment. P8 is appropriate insomuch as it represents, essentially, a functional definition of intrinsic value (see Appendix [55]). The most basic leads for evaluating this argument are represented by three questions: Who in the world possesses intrinsic value, what counts as fair treatment, and how should conflicts between competing interests be resolved?

An important consideration for the first question includes the existence of well-developed and robust reasons for acknowledging the intrinsic value of all individual vertebrate organisms (including all lions and all humans, Appendix). The risk of controversy rising from that reasoning ought to be minimal given that sociological evidence indicates widespread belief that vertebrates possess intrinsic value (Appendix). Hereafter, we refer to possessors of intrinsic value—to use jargon from scholarly ethics—as moral patients.

An important consideration for the second question—"what counts as fair treatment?”—is the notion that one should not frustrate the interests of a moral patient without adequate reason. That notion is a widely-appreciated variant of the law of equal liberty [56,57]. An adequate reason may require taking account of competing interests among moral patients whereby satisfaction of both interests is impossible. Such a circumstance can be evaluated, at least in part, with two follow-up questions: Is there asymmetry in vitalness of the competing interests, and can either of the competing interests be met by some other means?

To illustrate, consider the Maasai of southern Kenya and their ancient cultural interest to practise solemn ceremonial killing of lions to mark the passage of a boy into manhood. The competing interest is the lion’s interest to not be killed. The interest to live seems more vital than the interest to practise a particular ceremony. Moreover, the Maasai have demonstrated that their interest in this solemn ceremony can be met by practicing a less lethal adaptation of the ceremony [58].

Another tool for adjudicating competing interests is a particular gedanken-experiment, a variant of a thought experiment known as the veil of ignorance [57]. In this thought experiment, a group of denizens develop the rules and norms of society and do so without knowledge of the role each will have in that society. That is, each denizen is ignorant of what will become their personal socioeconomic status, religion, race, gender, etc. While the veil of ignorance and its variants have typically tended affairs that concern only humans, it is also appropriate to imagine a veil whereby a denizen is also ignorant of whether they will be human or nonhuman [49]. Agreeing to some particular adjudication of competing interests while behind the veil of ignorance is at least suggestive of fair adjudication.

In some cases, the preceding considerations will be insufficient. Consider, for example, a scenario where a lion is about to kill a member of some human’s immediate family; the only way to prevent the death of the family member is to kill the lion. The case is extreme because the competing interests are equally vital and cannot coexist. In extreme cases of this nature, relatedness may be the resolving consideration. Sparing the life of the one who is more closely related is reasonable, even though it is also tragic.

The preceding considerations are readily applicable to moral patients that possess interests, such as organisms. However, certain peculiarities arise in treating species and ecosystems—i.e., ecological collectives, the object of conservation concern—as moral patients because those kinds of things do not possess interests (Appendix, [18,49,55]). Tending those peculiarities here would take us from the central focus of this paper. Here, it suffices to say: Leaving unresolved the intrinsic value of an ecological collective, such as *Panthera leo*, the species, does not undermine the widely acknowledged value of preventing further diminishment of that species. However, leaving too much of the species’ value unspecified increases the risk of improper adjudication when the interest to conserve that species conflicts with some other (vital) interest.

This argument (P7, P8, and C4) leads to two general lessons (Table 1). First, account for the intrinsic value of humans and nonhumans who possess it. Doing so can be difficult, but no more so, in principle, than accounting for the intrinsic value of two or more humans with competing interests. Second, competing interests can be adjudicated, at least in part, by evaluating questions like: Is there asymmetry in the vitalness of the competing interests, and can either of the competing interests be met by some other means? Another useful guide for adjudication is the veil of ignorance thought experiment.

## 7. The Ethics of Animal Farming

A proponent of lion farming may intuit that: Many kinds of animals are farmed for many different reasons. Lions are not even the only kind of carnivore to be farmed. Red and Arctic fox and American mink, for example, are also farmed. Why should lion farming be viewed any differently than other forms of farming?

If one were to express that intuitive reasoning as a formal argument, it would be an argument by comparison (or contrast). (In our particular case, a comparison among different kinds of farming.) While this form of argument can be especially insightful, it is important to bear in mind that any two things—no matter how disparate—can be compared. And any two things—no matter how closely related—can be contrasted. As such, the requirement is to highlight a salient comparison or contrast. One method for identifying salient features is to construct an argument comprising one or more premises that make explicit (if not arguable) claims about, in this case, what constitutes an appropriate form of farming. For example:

- Premise 10 (P10). Farming that entails premature death of an animal is appropriate if: (1) The primary reason for farming is production of meat for human consumption, (2) the kind of animal used represents at least a relatively efficient means of producing meat, and (3) the animals’ wellbeing while alive is adequate.
- Premise 11 (P11). Lion farming is not characterised by those properties.
- Conclusion 5 (C5). Lion farming is not appropriate.

A critical point of evaluation for this argument pertains to the truth-value of P10. If P10 is appropriate, then P11 is certainly appropriate because lions are not raised for meat, nor are they an efficient means of meat production (because they are carnivores), and the argument would fail. The importance of efficient meat production is tied to concerns about the environmental impact of meat production [59].

A critic may think the criteria represented by P10 are too restrictive, or perhaps not restrictive enough. A thorough evaluation of P10 is beyond the scope of this paper. Nevertheless, we can say that thorough evaluation of the three criteria, 1–3, in P10 can be evaluated with principles represented by lesson 7 (Table 1). Evaluation of P10 may also require developing a subsequent argument, whereby one treats, for example, criterion 1 as the conclusion to some unstated argument:

- Conclusion 6 (C6). Farming that entails premature death of an animal is appropriate if the primary reason for farming is production of meat for human consumption.

The challenge is to discover and articulate the premises that would have to be true for that conclusion to follow. This leads to an important general lesson (Lesson 8 in Table 1): When the truth-value of a premise is in doubt—especially a normative premise—pull that premise from the argument, treat it as the conclusion to some other unstated argument, build and evaluate that argument.

The intuitive reasoning that introduced this Section 7 is associated with another broad lesson. For millennia, logicians have curated an encyclopaedia of logical fallacies—that is, forms of argumentation that are commonly employed yet fallacious and thus do not support conclusions. One of many such curations is Withey and Zhang (2016) [60]. These encyclopaedias of logical fallacies include various forms of fallacious comparison. The logical structure of a valid comparison continues to be an area of active research among logicians [61]. A narrow lesson is that argument by comparison—while valuable—can also be challenging.

The broader lesson is that encyclopaedias of logical fallacies can be a great aid in evaluating ethical arguments. Some logical fallacies are known colloquially, e.g., slippery slope, red herring, and appeals to authority. Others are no less important, but less appreciated outside the community of logicians and philosophers, e.g., fallacy of composition, the modal fallacy, and the fallacy of the undistributed middle.

## 8. Discussion

In this paper, we presented a set of arguments for and against lion farming that focused on a variety of reasons, including conservation, intrinsic value, and economics. The value of argument analysis in each case is, in part, to clarify precisely what insight (empirical or normative) needs to be taken into account.

While we evaluated some key concerns related to lion farming, we did not evaluate every concern. Some of these untended concerns include the cultural value of lion farming for traditional medicine in Africa and Southeast Asia or the sociopolitical history of South Africa. Lessons 5 and 6 (Table 1) would likely play a central role in evaluating arguments on those topics. Support for the commodification of lion bones might also rise from intuitions that the most honourable treatment of an exploited lion is to use it in the most complete and efficient means possible—including the use of their bones after having been trophy-hunted. Another consideration includes concern for the fate of captive lions should lion farmers abandon pursuit of the economic profit of lion farming—who would care for those lions then? Treatment of these and other issues are beyond the scope of this paper, which is not to have fully evaluated the case of farming lions with use of their bones, but rather to demonstrate a mode of discourse.

### 8.1. Ethical Pluralism

This mode of discourse lends itself to ethical pluralism. Ethical theory is vast, with at least three major frameworks (deontology, utilitarianism, and virtue ethics) and many more specific perspectives. More than a dozen formal ethical perspectives exist even within the realm of human–animal relationships [62]. With this diversity of perspective, a common modus operandi of applied ethics is to develop insight by confronting a real-world case with a particular ethical theory or perspective.

Without diminishing the value of that approach, argument analysis is valuable for not making any presupposition about any particular ethical framework or perspective. The presuppositions of argument analysis are limited to those surrounding the notions of soundness and validity, as those terms are understood by logicians [15]. As such, argument analysis is as morally pluralistic a tool as could possibly be.

This moral pluralism does not prevent argument analysis from being incisive with respect to particular ethical perspectives. To illustrate, an important perspective in human–animal relationships is that associated with care ethics, represented, for example, by reference [63]. One can evaluate any argument about lion farming from that perspective. As in any case, the form of evaluation is typically to scrutinise the truth-value of premises in the argument or to identify a missing premise whose appropriateness and inclusion would render the argument invalid. Alternatively, one can add to the list of existing arguments by constructing an argument afresh—an argument whose distinctive premises would be inspired by, in this case, care ethics. Using argument analysis in this way—to make incisive observations about a particular ethical perspective—does not obviate the value of the general lessons of Table 1.

### 8.2. Decision Making

**Figure 1:**
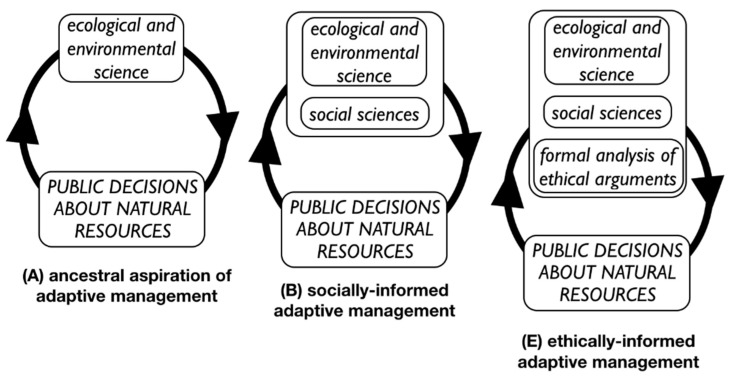
Evolution of public decision-making for natural resources. The upward-pointing arrowhead refers to evaluation of the consequences of a policy; the downward-pointing arrowhead refers to revision of policy based on the evaluation. Adapted from Reference [16].

The mode of discourse exemplified here would be of great benefit to decision-making in natural resource management. Its incorporation would represent a continuance of a gradually evolving process. A few decades ago—and according to certain principles of adaptive management [64]—decision-makers aspired to a process where decisions emerged from a presumed public good or goal and the best-available scientific knowledge (typically, ecological or environmental knowledge); the consequence of the decision was subsequently subject to scientific evaluation, leading to a revised and improved decision, whose consequences are also subjected to scientific evaluation. The process is a perpetual feedback loop that does not wait for final definitive answers from science (Figure 1).

That aspiration for decision-making has developed over the past few decades to also take account of the social sciences, which aim to understand what various stakeholder groups think about purported public goals and why. As before, public decisions are informed and revised by ever-emerging insight from social science, but the decision-making process does not wait for final or definitive insight. This evolved decision-making process can aptly be called socially-informed adaptive management (Figure 1).

The principles presented here—illustrated by concerns about farming lions—are most usefully seen as a key ingredient in the next evolution in adaptive decision-making, what might be considered ethically-informed adaptive management, which is simultaneously informed by insight from ecological science, social science, and ethical analysis [3,16]. As with prior forms of adaptive decision-making, decisions are not held off indefinitely, waiting for some final ethical insight. Rather, ethical insight is treated like scientific insight—they are all are part of a perpetual feedback loop, producing decisions that adapt to new insight as it becomes available.

### 8.3. Some Considerations on Implementation

The mode of discourse exemplified here may be implemented in various ways. It may begin with an analysis produced by experts and conveyed, for example, via a technical paper and subsequently evaluated (according to the principles of argument analysis) by stakeholders or decision-makers. It can emerge from stakeholders guided by a mediator with facility in applied ethics. In either case, the analysis described here is a first step in the process. The next steps could, for example, be for a decision-maker to vet the arguments and their evaluations with relevant stakeholders. While that vetting may lead to previously unrecognised points of agreement, the minimum standard for success is that the stakeholders agree that their understandings are well represented in the analysis. Afterward, the decision-maker can synthesise the insights and limitations of each argument toward the end of making a decision, as in, e.g., Reference [14].

Implementation of argument analysis is also informed by basic principles of moral psychology, the science aimed at understanding not how we ought to make ethical decisions, but rather biotic constraints and tendencies in how we actually make decisions. (Ethical and moral are synonymous, here.) Moral psychology indicates that moral judgments rise from rapidly-formed intuitions, and then, subsequently—if pressed to do so—one develops reasoned rationale for an intuited judgment [65,66]. These intuitions are also powerfully reinforced by the social groups with which we self-identify, increasing the difficulty of seeing alternative positions [67]. These are empirical claims supported by considerable evidence about how humans tend to make moral judgements. They are not normative judgments about how one ought to behave.

Those insights from moral psychology raise concern about motivated reasoning [65,66], jargon from psychology referring, in part, to an (unconscious) tendency to readily acknowledge evidence and arguments that favour one’s intuition and be reluctant to acknowledge evidence and arguments to the contrary. These are tendencies that are stronger in some people and on some occasions, and few humans are naturally free of the tendency. In argument analysis, that reluctance may be manifest as failure to see a genuine shortcoming of a premise, a gap in logic between the conclusion and a set of otherwise appropriate premises, or a premise that is appropriate and whose inclusion to the argument would undermine the argument.

An important response to concerns about motivated reasoning is to acknowledge that argument analysis is most powerful when one is (1) exploring a case about which one is genuinely unsure, (2) sincerely motivated to be better understood by another where prior attempts have fallen short, or (3) sincerely motivated to understand another’s contrary view. It is also helpful (4) to conduct argument analysis in an environment where a rich understanding of various countervailing views is readily available. Argument analysis can, of course, be used to simply promulgate a view. Even that motivation is most likely satisfied by carefully tending to 2, 3, and 4. These considerations do not obviate the vital importance of other practices of collaborative engagement (e.g., [67]).

The primary burden on the analyst (be they an expert analyst or a group of lay stakeholders) is to be honest in a very particular manner—that is, honest in relating their intuited moral judgment (about a policy) to a particular reason, from which a formal argument is built and analysed. This burden to honesty is opposed to connecting one’s intuited judgment to a reason (and subsequently, an argument) that is hoped to be persuasive, but is in fact a red herring and unrepresentative of one’s sincere concern [14]. The decision-maker has a different burden—that is, to be fair in making a decision given the available insight—scientific and ethical. What it means to be a fair decision-maker is discussed elsewhere [49].

Those burdens—to be an honest advocate and a fair adjudicator—do not distinguish ethically-informed decision-making from any other form of decision-making. Rather, its distinctive feature is adherence to formal analysis of ethical arguments. That adherence carries two benefits. First, argument analysis is especially effective for increasing the clarity and precision of a stakeholder position, which encourages the revelation of, for example, unstated premises that may undermine (or strengthen) support for an ethical conclusion or reveal an argument’s dependency on some logical fallacy [60].

A second benefit of adhering to argument analysis pertains to an ever-present threat to processes aimed at fair engagement of stakeholders—that is, the engagement of stakeholders with unequal political power [49]. Unequal distribution of political power can lead to tyrannical forms of democracy. The most important antidote for tyrannical forms of democracy (and populism) is to found public discourse not on intuited moral judgments, but rather on the evaluation of well-formulated reasons [68,69]. As such, unjustified inequalities of power among stakeholders would be neutralised to the degree to which argument analysis can be rigorously employed. This is not a recipe for conjuring a kind of utopia. Rather, the claim is that treating the rigorous application of argument analysis as a vital aspiration will lead to improved public decisions.

### 8.4. Conclusions

Publication of an analysis like this is not the end of the analysis, but rather a step in the process. The value of argument analysis is not its definitiveness, but rather its ability to produce clear and precise expressions of a position. Argument analysis tends to expose the limits of an argument. Those limits might be used to indict an advocate of an argument. But those limits might also be clues to previously unrecognised insight. An ethical argument in public discourse can be like a scientific hypothesis. A hypothesis is never proven true, it merely withstands more and more severe tests until someday it is toppled by a better understanding. Similarly, some ethical arguments—especially the ones associated with controversial public policies—might usefully be seen as provisional. This is not an endorsement of moral relativism; it is only intended to acknowledge moral fallibility.

Our analysis has implications for understanding binding international agreements pertaining to the relationship between humans and nonhumans. Except for some multilateral agreements among members of the European Union, the most powerful such agreements are primarily or exclusively motivated by conservation, not animal welfare. This is peculiar to the extent that animal welfare may be as deeply valued among nations and their citizens as conservation. Efforts to advance binding multilateral agreements focused on animal welfare are of significant value (e.g., Universal Declaration on Animal Welfare [70,71]). However, sets of agreements focused exclusively on conservation or animal welfare will fall short because the relationship between humans and nonhumans—especially at global scales—is an entangled nexus of concern for conservation and animal welfare. There is a need for developing a *Universal Declaration of Right Relationship Between Humans and Nonhumans*—a declaration that clearly articulates (1) values widely held by many nations as they pertain to conservation and animal welfare and (2) widely agreed upon principles for adjudicating conflicts that routinely arise when attempting to satisfy concern for the well-being of humans, individual nonhuman animals, species, and ecosystems. One challenge would be determining precisely what adjudicating principles are indeed widely held.

Closing on a note led by moral psychology, moral judgments do tend to be more intuitive than they are reasoned [72,73]. However, moral intuitions are not immutable. They develop—even if only slowly. Moral intuitions develop both within an individual when they mature, and they develop at the level of entire societies. An important basis for this development of intuition is public discourse surrounding various rationales for various intuitions—even if those rationales tend to be post hoc. The salient point is that the growth of ethical understanding is slow, but not intractable—much like the development of scientific understanding.

## Figures and Tables

**Table 1:** The analysis developed here—while motivated by an interest to evaluate the appropriateness of lion farming—leads to the identification of general lessons of value to any ethical analysis.

Lesson	Description
1 (Section 4)	A common misconception is that evaluating ethical arguments necessarily requires evaluating underlying, thorny and seemingly intractable ethical premises. Often enough, however, an ethical argument fails because one or more empirical premises do not hold—precluding the need to evaluate the ethical premises.
2 (Section 4)	Uncertainty about the truth-value of a premise can be built into the verbiage of a premise and subsequently the conclusion. See, for example, the argument represented as P1′, P2, and C1′.
3 (Section 4)	Invoking the precautionary principle is of limited value for overcoming an impasse, unless it is accompanied by careful analysis of all the various harms that might be judged as irreparable.
4 (Section 5)	Attend not only to the consequences of an action, but also to concerns about whether the consequences can be justified given the proffered means.
5 (Section 5)	Express end-goals with sufficient precision. Specificity aids evaluating (1) merit of the end-goal, (2) alternative means of realising the end-goal, and (3) whether the proffered means would lead substantively (or only trivially) to the end-goal. For example, “create new jobs” is a vague end-goal that might be expressed more precisely as “decrease unemployment rate in some region to less than 5%”.
6 (Section 6)	Account for the intrinsic value of humans and nonhumans that possess it. Doing so can be difficult, but no more so, in principle, than accounting for the intrinsic value of humans with competing interests. See Section 6 for details.
7 (Section 6)	Competing interests can be adjudicated, at least in part, by evaluating questions like: Is there asymmetry in vitalness of the competing interests, and can either of the competing interests be met by some other means? Another useful guide for adjudication is the veil of ignorance thought experiment. See Section 6 for details.
8 (Section 7)	When the truth-value of a premise is in doubt—especially a normative premise—pull that premise from the argument, treat it as the conclusion to some other unstated argument, build and evaluate that argument.
9 (Section 7)	Argument by comparison—while valuable—can also be challenging.
10 (Section 7)	Encyclopaedias of logical fallacy can be a great aid to evaluating ethical arguments.

**Table 2:** Science is characterised by the quantitative expression of likelihood. Yet, deductive reasoning is essentially qualitative. This table—used by the Intergovernmental Panel on Climate Change (IPCC) [43]—is a useful bridge between the qualitative and quantitative (Section 4.4).

Qualitative Expression	Quantitative Expression
Virtually certain	99–100% probability
Extremely likely	95–100% probability
Very likely	90–100% probability
Likely	66–100% probability
About as likely as not	33–66% probability
Unlikely	0–33% probability
Very unlikely	0–10% probability
Extremely unlikely	0–5% probability
Exceptionally unlikely	0–1% probability

## Appendix A. A Primer on Intrinsic Value

This Appendix is adapted slightly from its source, which is Appendix 3 of Reference [18]. An object is instrumentally valuable if valuable as a means to some other end and intrinsically valuable if valuable beyond its instrumental value or valuable for its own sake [74]. While succinct definitions of intrinsic value tend to be abstract and easily misconstrued, the implication of something possessing intrinsic value is straightforward: If something possesses intrinsic value, then we have an obligation to treat it with respect or fairly and with at least some concern for its wellbeing or interests [56]. As such, it is wrong to harm an intrinsically valuable entity without an adequate reason.

The formal ethical reasoning supporting the claim is also robust. One of the more important lines of reasoning begins with the supposition that humans possess intrinsic value because we have interests (e.g., to avoid pain and to flourish). Given that supposition, it follows that any entity with such interests would also possess intrinsic value. Because all vertebrate organisms possess those interests, they also possess intrinsic value. The force and universality of that reasoning is indicated by the principle of ethical consistency, i.e., treat others as you would consent to be treated in the same position (for a general review, see Reference [75]; for applications to nonhumans, see References [76,77,78]; see Nussbaum (2006) [53] for a similar application of a traditionally anthropocentric ethic to nonhumans). Most human cultures are undergirded by some variant of this principle (e.g., Golden Rule). The intrinsic value of at least some nonhuman portions of nature is also widely appreciated—as reflected by sociological evidence [79] and many governments [55].

Because all living things have an interest to flourish, a case has been made that all living things possess intrinsic value—a position known as biocentrism [80,81]. The argument presented above does not require acknowledging biocentrism; it only requires accepting a narrower and uncontroversial belief, sentientism, which acknowledges that sentience imbues a thing with intrinsic value. Lions and other mammals of the Order Carnivora (which, for example, contains dogs and cats) are sentient and thus—according to sentientism—possess intrinsic value.

While those considerations pertain to the intrinsic value of organisms, different reasoning is required to consider the intrinsic value of ecological collectives, such as populations, species, and ecosystems. One line of reasoning would be that ecological collectives are normally homeostatic, resilient, and interconnected and that those properties imbue them with intrinsic value [82]. Some, but not all, ecologists believe that ecological collectives are *not* characterised by those properties [83,84,85,86]. Nevertheless, whether an ecological collective possesses those traits is not entirely a scientific question but is in an important sense a metaphysical question (to illustrate: Describing the interconnectedness of a system is usefully considered a purely scientific endeavour—however, see Putnam, 2004 [87]; but judging whether those interconnections are sufficiently intimate for the system to qualify, for example, as an organism involves a significant metaphysical considerations, e.g., [88,89]. As such, it is at least partially relevant that many (if not most) people believe that “nature possesses a delicate balance that is easily upset by humans” [90].

A second line of thinking (developed by Leopold, 1949 [82]) also supports the intrinsic value of ecological collectives. That line of thinking begins with the supposition that we humans, along with ecological collectives, are members of the same biotic community. In sharing community membership, and by extending the moral principles that apply to human communities, we ought to treat ecological collectives with respect.

Taking for granted that individual animals and ecological collectives both possess intrinsic value leads to an unresolved concern that is aptly represented as an incomplete argument:

- P1. Individual lions possess intrinsic value.
- P2. *Panthera leo*, the species, possesses intrinsic value.
- P3. ???
- C. When the intrinsic values of P1 and P2 conflict, the intrinsic of P? supersedes.

The unresolved nature of this argument is conveyed in Section 1.
